# New Hydrophilic Derivatives of Lasalocid and Their Complexes with Selected Metal Cations

**DOI:** 10.3390/molecules28135114

**Published:** 2023-06-29

**Authors:** Monika Papsdorf, Radosław Pankiewicz

**Affiliations:** Department of Enviromental Physicochemistry, Faculty of Chemistry, Adam Mickiewicz University, Poznan Uniwersytetu Poznańskiego 8, 61-614 Poznań, Poland

**Keywords:** lasalocid, polyether antibiotics, metal complexes, spectroscopy, theoretical calculations

## Abstract

Two new esters of lasalocid, that are more hydrophilic, with glucose (LasGlu) and xylitol (LasX), have been synthesized, and their complexation of monovalent cations has been studied by various spectrometric and spectroscopic methods, such as ESI mass spectrometry, ^1^H, ^13^C NMR and FT-IR. Analyses of the results confirmed the synthesis of new esters with good yields. In order to carry out further studies, it was necessary to purify them using “flash“ liquid chromatography. It was confirmed that the newly obtained molecules, as well as their complexes with lithium, sodium and potassium cations, were stabilized by a strong system of intramolecular hydrogen bonds. It was found that the hydroxyl groups of esters derived from xylitol and glucose were also involved in the complexation of cations. The results of the PM6 semiempirical calculations permitted determination of the heat of formation (HOF), and visualization of the structure of the new esters and their complexes with the cations studied. All computation results are in agreement with the spectroscopic data.

## 1. Introduction

Carboxylic polyether ionophores make a large group of natural compounds, produced by various species of Streptomyces. The terms of ion-carrying antibiotic and ionophore were first used by Presman et al. [1], who described the mechanism of action of valinomycin employing its ability to selectively bind potassium cations and transport them through lipid membranes. Ionophores can be classified according to different features, e.g., the origin (natural, synthetic), chemical structure (cyclic, non-cyclic, carboxylic) or ion transporting method (conveyor and channel forming—called pseudoionophore), and their type (monovalent and divalent) [2].

Ionophore antibiotics belong to the class of naturally-occurring materials showing diverse biological activity that are widely used in the animal husbandry industry and in veterinary science as, e.g., feed additives and medicines. Lasalocid, salinomycin and monensin are classified as the natural antibiotics most often used in poultry and cattle breeding. They are applied as feed additives to stimulate weight gain, as well as to prevent and treat coccidiosis [3,4,5,6].

The above compounds have a non-cyclic structure, but the presence of a carboxyl group at one end of the molecule, and a hydroxyl group at the other, permits the formation of hydrogen bonds within the molecule. In the presence of different metal cations, these compounds may form host–guest assemblies. An ionophore may comprise in its molecular scaffold both the lipophilic, as well as the hydrophilic, counterparts (–OH, C=O, C–O–C), which permits complexation of metal cations inside the lipid membrane [7].

Even slight structural modifications [8,9,10,11,12,13] of the antibiotic molecules may result in changes to the structure of the complex, which affects the ability of the ionophore to bind metal ions and transport them through the cell lipid layers. For biologically active compounds, such changes may have an impact on their antimicrobial properties [14,15].

Currently, particular interest is paid to ionophore antibiotics, because of their verified anticancer activity, including that against multidrug resistant cancer cells. The pioneers in the study of ionophore antibiotics as anti-cancer agents were Gupta et al. [16], who screened about 16,000 compounds to find that ionophore antibiotics are extremely effective in killing epithelial cancer cells. Salinomycin, and the structurally related nigericin, exhibit selective toxicity to cancer stem cells (CSC), and are about 100 times more potent compared to the cytotoxic paclitaxel used. This landmark study has stimulated many further studies and questions. Lasalocid may also be useful in the search for new potential chemotherapeutic agents and in the studies aimed at better understanding the biochemical processes taking place in cancer cells [16,17,18].

Ionophore antibiotics are promising compounds showing firm antitumor and anti-CSC activity. However, resistance associated with drug transportation potentially limits their use to CSC populations that do not have significant drug-release capacity. Further research is required to determine which subsets of cancer stem cells may be susceptible to the cytotoxicity of ionophore antibiotics, and profiling the tissues of the stem cells from which they originate to obtain valuable input data has been proposed [19].

So far, modifications of natural lasalocid have been focused mainly on the creation of lipophilic derivatives containing alkyl chains or aromatic rings in their structure. Much attention has also been devoted to the synthesis of derivatives containing polyether structures, such as oxaalkyl [10,20,21], hydroxyoxaalkyl [9,22,23], ethylene glycol [8,24] and crown ether [25], to increase the ability to form complexes of alkali metal cations and the solubility of the complex in the lipid membrane [19]. However, a good ion carrier should be characterized not only by the ease of forming stable complexes, but also by the ease of releasing complexed ions. For this reason, the synthesis of new derivatives of lasalocid with increased hydrophilicity was undertaken. In this study, new esters of lasalocid with glucose (LasGlu) and xylitol (LasX) have been synthesized, and their ability to form complex with Na^+^, K^+^ and Li^+^ cations has been examined. By means of HPLC and NMR methods, the identities of the two ester derivatives of lasalocid acid have been confirmed. ESI-MS and FT-IR studies provided evidence that LasGlu and LasX esters form complexes with monovalent metal ions at a molar ratio of 1:1.

## 2. Results and Discussion

### 2.1. ESI Mass Spectrometry

**Figure 1:**
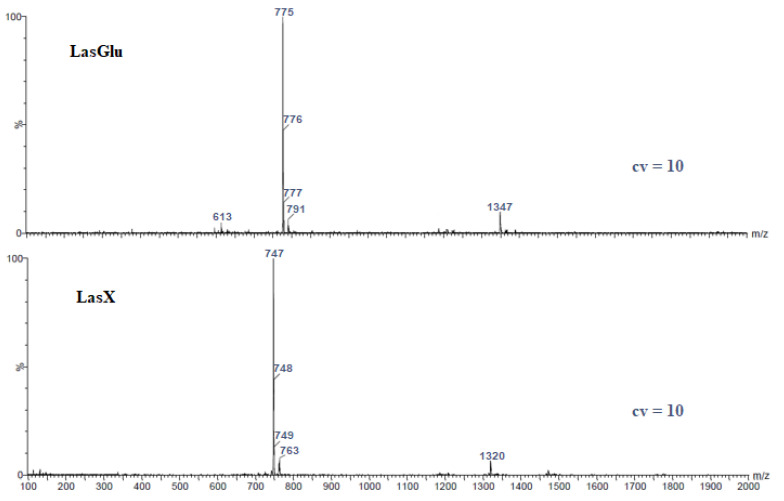
ESI mass spectra of lasalocid esters.

Figure 1 shows LasGlu and LasX mass spectra. The spectra were recorded using a very gentle ionization method—electrospray ionization (ESI)—additionally, a very low cone voltage (10 V) was used. Thus, while it was possible to obtain very clear molecular peaks, we did not actually observe signals from the fragmentation product. This approach allows us to observe molecular peaks from the formed complexes.

In the spectrum in Figure 1 (LasGlu), the most intense signal at *m*/*z* = 775 is assigned to the LasGlu complex with sodium cations. Accordingly, weaker signals at *m*/*z* = 776 and *m*/*z* = 777 are assigned to isotopic peaks originating from ^13^C carbon atoms incorporated in the ester molecule. We also observe very weak signals at *m*/*z* = 791 assigned to the LasGlu complex with the K^+^ cation. On the other hand, a very weak signal at *m*/*z* = 1347 suggests the formation, during the synthesis, of a by-product in the form of a double-substituted glucose molecule with lasalocid, which complexes a single sodium cation. The very weak signal at *m*/*z* = 613 is attributed to the complex of lasalocid acid with sodium cation, probably a fragmentation ion.

A similar situation is observed in the mass spectra of lasalocid ester with xylitol (Figure 1 (LasX)). In the analyzed spectrum, the most intense signal at *m*/*z* = 747 is assigned to the LasX complex with sodium cations, while the weaker signals at *m*/*z* = 777 and *m*/*z* = 778 are assigned to isotopic peaks coming from ^13^C carbon atoms incorporated in this molecule. We also observe very weak signals at *m*/*z* = 763 assigned to the LasX complex with the K^+^ cation. On the other hand, a very weak signal at *m*/*z* = 1320 suggests the formation, as above, of a by-product in the form of a double-substituted xylitol molecule with lasalocid, which complexes a single sodium cation. The main peaks in the ESI mass spectra of the lasalocid complexes are summarized in Table 1.

The analyzed data obtained from mass spectrometry clearly confirm the identity of the synthesized chemical compounds. It is worth noting that the samples for analysis were prepared without the addition of appropriate sodium and potassium salts. The complexed cations most likely come from the glassware used during the synthesis and purification process. This result proves the great ability of the obtained lasalocid derivatives to complex monovalent metal cations.

### 2.2. NMR Measurements

**Figure 2:**
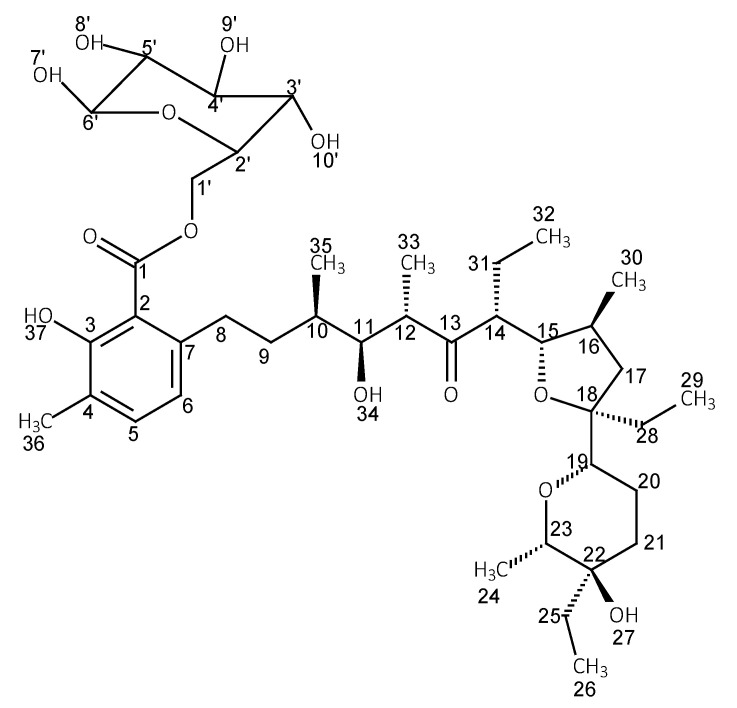
Structure of lasalocid ester with glucose.

**Figure 3:**
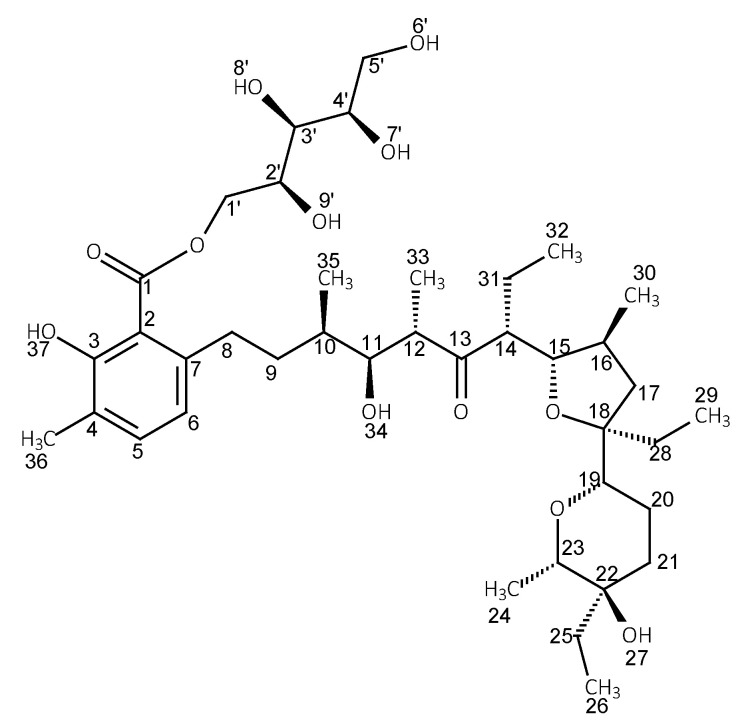
Structure of lasalocid ester with xylitol.

The ^1^H and ^13^C NMR data of LasH in chloroform and its esters with glucose and xylitol are collected in Table 2, Table 3 and Table 4. Table 3 and Table 4 additionally show the chemical shifts of β-d-glucose and xylitol. The table has been divided into three smaller ones to ensure better readability. The structures of the corresponding esters along with the numbering of the atoms are shown in Figure 2 and Figure 3.

In the ^1^H NMR spectra of LasH, the signals of protons of the OH groups are found separately at 11.20, 3.40 and 2.40 ppm. The signal at 11.20 ppm is assigned to the O_37_H proton of the phenolic group involved in the middle strong intramolecular hydrogen bond. The positions of the proton signals of the other OH groups indicate that they are involved in relatively weak intramolecular hydrogen bonds.

In the ^1^H NMR spectra, the signals from the protons at C_8_ are split. This proves the inhibition of the rotation of the salicylic part of the molecule (the “head”) in relation to the rest of the chain. In addition, the splitting of the signals assigned to C_17_H, C_20_H and C_30_H indicates a restriction in the movements of the chain itself. It is most likely due to the formation of a head-to-tail hydrogen bond, and a series of other intramolecular hydrogen bonds, that stabilize the conformation of the entire molecule, which is confirmed by semiempirical and DFT calculations.

Analyzing the data in Table 3, we can see a significant chemical shift in C_1′_H in both ^1^H NMR and ^13^C NMR spectra, of 0.81 ppm and 5.06 ppm, respectively, which proves the formation of an ester bond with lasalocid, as these are the atoms located directly next to it. Similar conclusions can be drawn by analyzing the data presented in Table 4; the chemical shifts of C_1′_H are clearly visible. It also proves the formation of the corresponding ester.

In the ^1^H NMR spectrum of LasGlu, we observe a clear shift of the signal assigned to O_37_H in the phenolic group towards stronger fields by 1.17 ppm.

This proves a weakening of this hydrogen bond. A similar chemical shift is observed for C_11_H, which indicates a change in the chemical environment, most likely caused by a change in the arrangement of the nearby hydrogen bonds. The signal from C_31_H was also averaged, which proves the increased mobility of the polyether chain. The other signals from protons, as in the light of the LasH molecule, remain cleaved, indicating that the rotation of the salicylic part is blocked. The same locking of rotation can be read from the analyzed LasX spectra.

### 2.3. FT-IR Measurements

**Figure 4:**
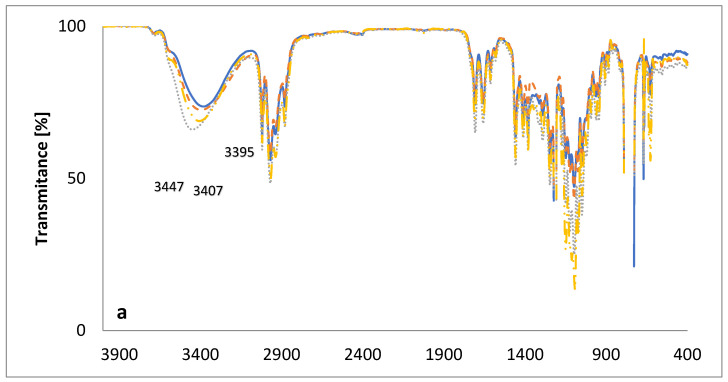

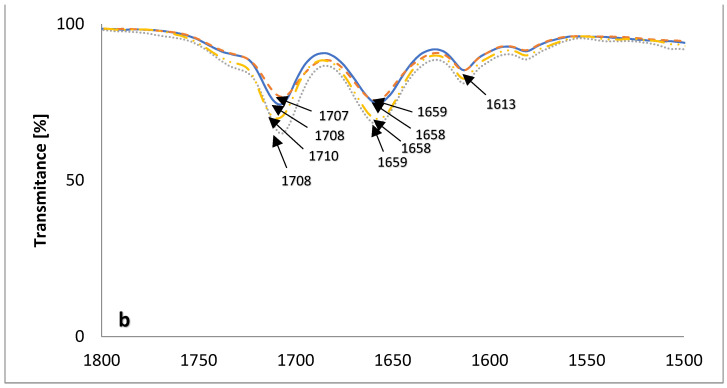
The FT-IR spectra of (—) LasGlu and its 1:1 complexes with cations: (– –) Li^+^; (⋯) Na^+^; (-··-) K^+^; (**a**) 4000–400 cm^−1^; (**b**) 1800–1500 cm^−1.^

**Figure 5:**
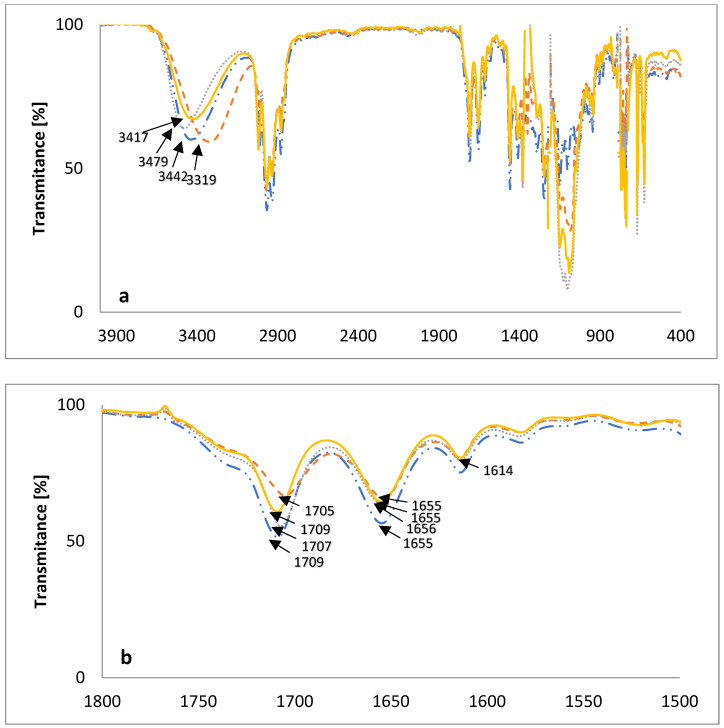
The FT-IR spectra of (—) LasX and its 1:1 complexes with cations: (– –) Li^+^; (⋯) Na^+^; (-··-) K^+^; (**a**) 4000–400 cm^−1^; (**b**) 1800–1500 cm^−1.^

The FT-IR spectra, in the mid-infrared region of the new lasalocid esters with glucose (LasGlu) (Figure 4) and xylitol (LasX) (Figure 5), and their 1:1 complexes with monovalent cations, are given in Figure 4 and Figure 5, respectively.

The bands assigned to the ν(OH) stretching vibrations of the hydroxyl groups in the spectrum of LasGlu (Figure 4a) appear as one broad band at 3382 cm^−1^. The appearance of this one band means that all hydrogen bonds in this molecule have similar strengths. The same bands in LasGlu complexes are slightly shifted to higher wavenumbers, 3395 cm^−1^ for 3447 cm^−1^ for LasGlu–Na^+^ and 3407 cm^−1^ for LasGlu–K^+^. This means that the formation of a complex with a cation slightly weakens intramolecular hydrogen bonds. The greatest weakening was observed for the Na^+^ cation, and the smallest for the Li^+^ cation.

Figure 4b shows the same spectra on an extended scale in the 1800–1500 cm^−1^ region. A comparison of the spectra of lasalocid ester and its complexes with Li^+^, Na^+^ and K^+^ cations shows only minor changes. In the spectrum of LasGlu, the maximum v(C=O) stretching vibration of the ketone group is observed at 1708 cm^−1^, whereas in the spectra of the complexes, it is shifted to 1707 cm^−1^ for LasGlu–Li^+^ and 1710 cm^−1^ for LasGlu–Na^+^, or not shifted for LasGlu–K^+^, indicating no or weak interactions of this ketone group with cations studied.

For LasX complexes with the same cations (Figure 5), we have a more-or-less similar situation, except for the complex with lithium cation.

In the spectrum of LasX (Figure 5a), the band assigned to the ν(OH) stretching vibrations of the hydroxyl groups appears as a singlet at 3442 cm^−1^, which means that all hydrogen bonds in this molecule have similar strengths. The same bands in LasX complexes are slightly shifted to higher wavenumbers for LasGlu–Na^+^—3479 cm^−1^ or lower ones for LasGlu–K^+^, which implies only slight changes in the arrangements of intramolecular hydrogen bonds. The situation is slightly different for the lithium cation complex. In this complex, the change is significant, as the absorption maximum of the corresponding band is shifted by as much as 123 cm^−1^ towards lower wavenumbers. This may indicate a strong interaction of the hydroxyl groups with the cation. We did not have such a situation with LasGlu complexes.

Figure 5b shows the same spectra on an extended scale in the 1800–1500 cm^−1^ region. A comparison of the spectra of lasalocid ester, and its complexes with Na^+^ and K^+^ cations, shows only minor changes. In the spectrum of LasX, the maximum ν(C=O) stretching vibration of the ketone group is observed at 1709 cm^−1^, whereas in the spectra of the complex with sodium, it is very slightly shifted to 1707 cm^−1^, or not shifted for LasGlu–K^+^, indicating no or weak interactions of this ketone group with the cations studied. In the spectrum of the lithium cation complex, we observe a shift in the absorption maximum towards lower wavenumbers by 4 cm^−1^. This shift is slightly larger than those of the other cations, but still small compared to those of the other lasalocid acid esters studied earlier [9,11]. This shows that, in the case of such strongly hydrophilic derivatives of lasalocid acid (LasGlu and LasX), the keto group is only slightly involved in the formation of the complex. This conclusion has been corroborated by the results of DFT calculations.

### 2.4. PM6 and DFT Study

The heats of the formation of LasGlu and LasX and their complexed and uncomplexed species with various monovalent cations are collected in Table 5. These data show that the formation of complexes between the studied esters and cations is energetically favorable.

A lower ΔHOF value means a higher energy gain obtained from cation complexation. In general, for the complexes of both esters, we observed a similar relationship: energetically, the complexation of the sodium cation is preferred, followed by the complexation of the lithium cation. The heat of complex formation with K^+^ is more than half lower than the heat of complex formation with Na^+^, which indicates that both LasGlu and LasX will preferentially form complexes with the sodium cation. This is probably related to the size of the formed cavity in the host ester molecule. Of course, the ligand structure is only pseudocyclic, and the size of the cavity is not theoretically limited, but intramolecular hydrogen bonds play an important role in stabilizing the structure.

A slightly different form of the LasX–Li^+^ complex found on the basis of the analysis of FT-IR spectra is not clearly reflected in the change of its ΔHOF value.

**Figure 6:**
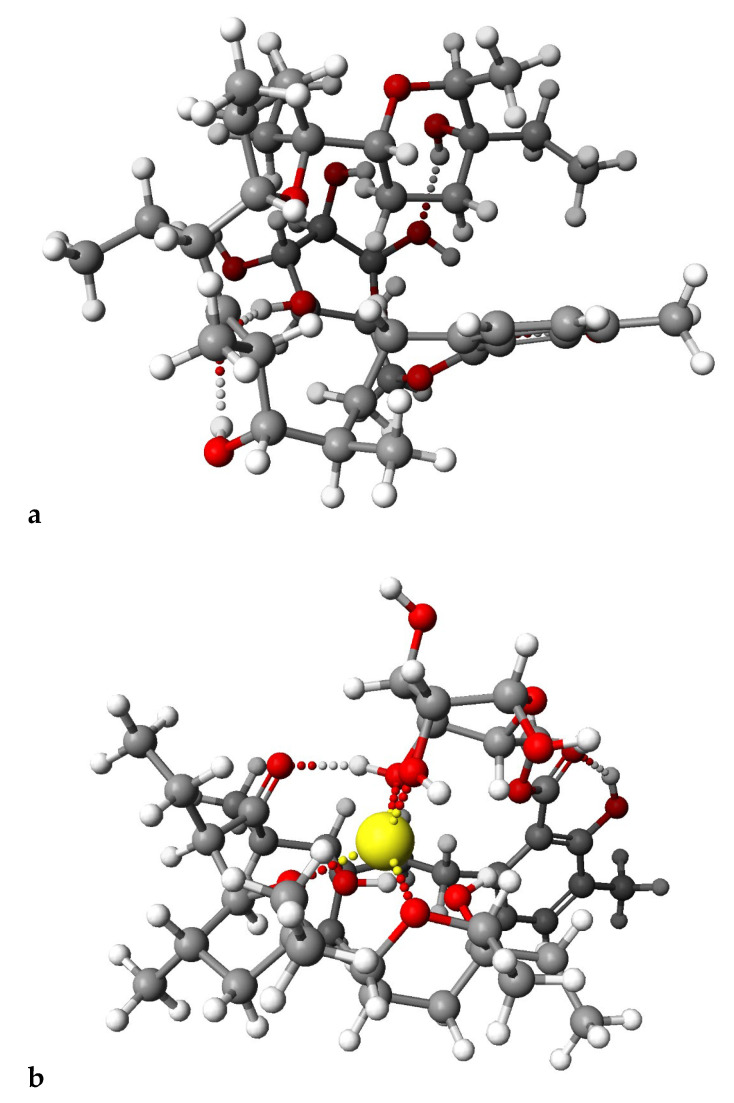
Calculated (DFT) structures of the LasGlu (**a**) and its 1:1 complex Li^+^ cation (**b**).

**Figure 7:**
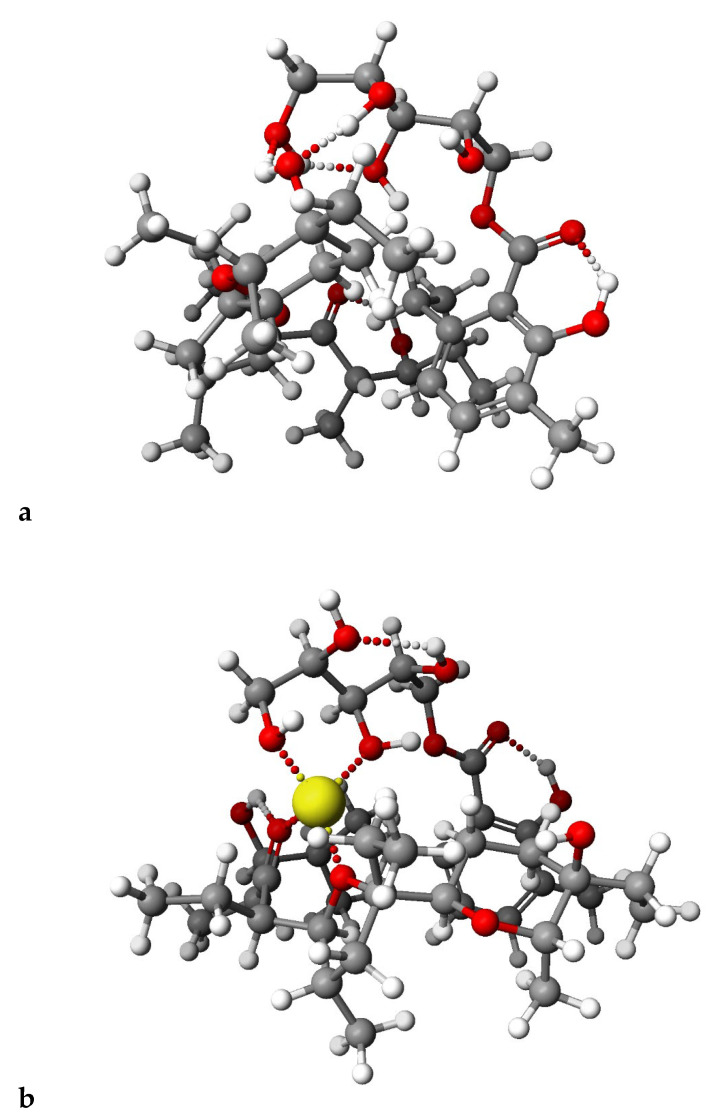
Calculated (DFT) structures of the LasX (**a**) and its 1:1 complex Li^+^ cation (**b**).

Figure 6 and Figure 7 presented calculated by DFT methods structures of LasGlu and LasGlu–Li^+^ (Figure 6) and LasX and LasX–Li^+^.

For both esters, we observed the presence of strong intramolecular hydrogen bonds, even after complexation of the cation, which is in agreement with the data obtained from the spectroscopic analysis. However, the arrangement of these bonds, as well as the conformation of the molecule, undergo some changes, which is in agreement with the results of analysis of ^1^H NMR spectra, showing that the splitting of the signal assigned to C_31_H proton disappears.

## 3. Materials and Methods

### 3.1. Preparation of Lasalocid Acid

Lasalocid acid was prepared from lasalocid sodium salt, which is one of the ingredients of the animal feed additive with the trade name Avatec. Initially, 250 g of the feed additive was pre-extracted in 2 L of hexane in a Soxhlet apparatus for 12 h to remove dyes and other impurities. Then, proper extraction in 2 L of methylene chloride in a Soxhlet apparatus for the next 12 h was performed, to obtain the sodium salt of lasalocid.

Lasalocid sodium salt (3.0 g) was dissolved in dichloromethane (50 mL) and transferred to a separatory funnel containing 50 mL of an aqueous solution of sulfuric acid, pH = 1.5. After intensive shaking, the organic layer containing lasalocid (LAS) was washed three times with distilled water. Subsequently, dichloromethane was evaporated under reduced pressure to dryness giving LAS (2.5 g).

### 3.2. Preparation of the Glucose Ester (LasGlu) and Xylitol Ester (LasX)

Lasalocid (0.005 mol) as well as glucose or xylitol (0.005 mol), was dissolved in 50 mL of pyridine. The reaction mixture was stirred intensively for 30 min. After that, 0.005 mol of 1,3-dicyclohexylcarbodiimide (DCC) was added to the reaction mixture, which was stirred overnight at ambient temperature. The precipitated dicyclohexylurea was filtered off, then concentrated under reduced pressure. Purification on a silica gel column using a CombiFlash NEXGEN 300+ system (Teledyne ISCO) (0 → 30% CH_2_Cl_2_/acetone) gave the product as a fluff.

### 3.3. Preparation of Complexes

The sources of Li^+^, Na^+^, K^+^ cations necessary for complex formation were LiClO_4_, NaClO_4_ and KClO_4_ (Sigma-Aldrich, St. Louis, MO, USA). Solutions of the complexes were then obtained by dissolving the appropriate salt (0.01 mol dm^−3)^ together with LasGlu or LasX in acetonitrile at the ratios 1:1. The mixture was stirred for about 1 h at ambient temperature. Acetonitrile was of spectroscopic grade. All preparations and transfers of solutions were carried out in a carefully dried glovebox.

### 3.4. ESI MS Measurements

The electrospray ionization (ESI) mass spectra were recorded on a Waters/Micromass ZQ mass spectrometer. The measurements were performed for the solutions of LasGlu and Las X (5 × 10^−4^ mol dm^−3^). The samples were prepared in dry acetonitrile, and were infused into the ESI source using a Harvard pump, at a flow rate of 20 mL min^−1^. For the standard ESI mass spectra, the cone voltage was 30 V. The source temperature was 120 °C and the desolvation temperature was 300 °C. Nitrogen was used as the nebulizing and desolvation gas, at flow-rates of 100 and 300 dm^3^ h^−1^, respectively. The mass range for ESI experiments was from *m*/*z* = 100 to *m*/*z* = 1300.

### 3.5. NMR Measurements

The NMR spectra were recorded on a BRUKER Avance III HD (^1^H NMR at 400.2 MHz and ^13^C NMR at 100.6 MHz) magnetic resonance spectrometer. The ^1^H NMR spectra are described in chemical shifts downfield from TMS, using the respective residual solvent peak as the internal standard (CDCl3 δ 7.26 ppm). The ^13^C NMR spectra are described in chemical shifts downfield from TMS using the respective residual solvent peak as the internal standard (CDCl3 δ 77.16 ppm). The line broadening parameters were 0.5 or 1.0 Hz, while the error of chemical shift value was 0.01 ppm. The ^1^H and ^13^C NMR signals were assigned independently for each species using one-or two-dimensional (COSY, HMQC) spectra.

### 3.6. FT-IR Measurement

All samples were measured in chloroform solution. The mass of each sample was 10 mg. The FT–IR spectra were recorded using a IFS 66/s FT-IR spectrophotometer from Bruker, equipped with an MCT detector (125 scans, resolution 2 cm^−1^).

### 3.7. Theoretical Calculations

PM6 semiempirical calculations were performed using the Scigress FJ2.6 (EU 3.1.9) program (Fujitsu, Tokyo, Japan). In all cases, full geometry optimization was carried out without any symmetry constraints. The DFT calculations were performed using the GAUSSIAN 16 package [26]. The geometries were optimized according to Becke’s three-parameter hybrid method with the Lee, Yang and Parr correlation functionals (B3LYP) [27] and 6-311G(d) basis set.

## 4. Conclusions

Lasalocid, thanks to its structure, forms a strong network of intramolecular hydrogen bonds directed to the inside of the molecule. That is why, although its molecules contain numerous hydrophilic groups, they are strongly hydrophobic.

To reduce the hydrophobicity of the molecule, we carried out esterification with strongly hydrophilic molecules—glucose and xylitol. After some searching, it was possible to select the appropriate solvent (pyridine) for the synthesis, in which all the substrates dissolved. The identity of pure LasGlu and LasX ester molecules was confirmed (after purification using flash chromatography). Both new molecules maintained the properties of the original ionophore. They were still hydrophobic and dissolved well in chloroform. They also effectively complexed cations, such as Li^+^, Na^+^ and K^+^. On the basis of quantum chemical calculations (PM6), we can conclude that the preference for complexing cations is ordered as follows: Na^+^ > Li^+^ > K^+^.

## Figures and Tables

**Table 1:** The main peaks in the ESI mass spectra of the lasalocid complexes at cone voltage 10 V.

Ester	Main Peaks (*m*/*z*)
LasGlu	613 (w), 775, 776, 777 (w), 791 (w), 1347 (w)
LasX	747, 748, 749 (w), 763 (w), 1320 (w)
(w) weak signal.	

**Table 2:** ^1^H NMR and ^13^C NMR chemical shifts (ppm) of LasGlu and LasX in chloroform.

No. of Atom	LasH		LasGlu		LasX		Δ LasGlu		Δ LasX	
	^1^H	^13^C	^1^H	^13^C	^1^H	^13^C	Δ^1^H	Δ^13^C	Δ^1^H	Δ^13^C
1	–	171.37	–	175.9	–	172.23	–	4.5	–	0.86
2	–	111.54	–	117.5	–	111.61	–	6.0	–	0.07
3	–	160.29	–	163.51	–	160.93	–	3.2	–	0.64
4	–	124.08	–	123.42	–	124.28	–	−0.7	–	0.2
5	7.13	134.99	7.16	132.33	7.17	135.32	0.03	−2.7	0.04	0.33
6	6.63	121.8	6.87	119.74	6.66	121.77	0.24	−2.1	0.03	−0.03
7	–	143.43	–	144.4	–	143.72	–	1.0	–	0.29
8	2.81; 3.00	33.75	2.90; 3.87	34.05	2.66; 3.10	34.58	0.09; 0.87	0.3	0.15; 0.10	0.83
9	2.19	35.38	2.25	34.83	2.48	35.24	0.06	−0.6	0.29	−0.14
10	1.76	33.95	1.99	34.0	1.64	35	0.23	0.0	−0.12	1.05
11	3.47	70.56	5.29	71.42	3.46	71.1	1.82	0.9	−0.01	0.54
12	2.9	48.99	2.94	49.51	2.83	49.63	0.04	0.5	−0.07	0.64
13	–	215.28	–	219.90	–	216.04	–	4.6	–	0.76
14	2.71	55.16	2.87	55.93	2.6	54.97	0.16	0.8	-0.11	−0.19
15	3.79	84.51	3.83	83.45	3.77	84.52	0.04	−1.1	-0.02	0.01
16	~1.6	36.1	~1.6	34.47	~1.6	36.71	0	−1.6	0	0.61
17	1.50; 1.82	39.22	1.45; 1.88	38.22	1.50; 1.85	38.56	0.05; 0.06	−1.0	0; −0.03	−0.66
18	–	71.43	–	76.42	–	71.58	–	5.0	–	0.15
19	3.55	72.56	3.63	69.11	3.69	73.42	0.08	−3.5	0.14	0.86
20	1.45; 1.66	20.64	1.43; 1.6	20.18	1.45; 1.7	19.79	−0.02; −0.06	−0.5	–; 0.04	−0.85
21	1.26	29.86	1.93	31.57	1.62	29.85	0.67	1.7	0.36	−0.01
22	–	85.8	–	87.83	–	87.49	–	2.0	–	1.69
23	3.74	~77	3.76	76.42	3.81	77.71	0.02	–	0.07	–
24	1.16	14.02	0.76	13.83	1.15	14.03	−0.40	−0.2	−0.01	0.01
25	1.55	30.61	1.26	31.51	2.17	30.71	−0.29	0.9	0.62	0.1
26	0.84	6.39	0.82	6.82	0.94	6.55	−0.02	0.4	0.10	0.16
27	~2.4	–	4.08	–	2.23	–	1.68	–	−0.17	–
28	~1.4	29.3	1.75	30.35	1.15	30.02	−0.35	1.1	0.25	0.72
29	0.79	8.54	0.74	9.74	0.98	9.34	−0.05	1.2	0.19	0.8
30	1	15.99	1.01	15.47	1.03	16.06	0.01	−0.5	0.03	0.07
31	1.40; 1.87	17.91	1.88	16.33	1.46	16.83	1 signal	−1.6	1 signal	−1.08
32	0.84	12.53	0.78	12.61	0.94	13.05	−0.06	0.1	0.10	0.52
33	0.9	13.49	1.13	13.62	0.92	13.41	0.23	0.1	0.02	−0.08
34	~3.4	–	~3.4	–	~3.4	–	0	–	0	–
35	0.9	12.37	0.91	12.64	1.07	12.88	0.01	0.3	0.17	0.51
36	2.19	15.87	2.03	16.31	2.21	15.91	−0.16	0.4	0.02	0.04
37	11.2	–	10.03	–	11.5	–	−1.17	–	0.30	–

**Table 3:** ^1^H NMR and ^13^C NMR chemical shifts (ppm) of Las Glu (glucose part) in chloroform.

No. of Atom	Glu		LasGlu			
	^1^H	^13^C	^1^H	^13^C	Δ^1^H	Δ^13^C
1′	3.57	61.09	2.76	56.03	−0.81	−5.06
2′	3.4	72.98	3.83	71.5	0.43	−1.48
3′	3.1	72.25	3.66	71.5	0.56	−0.75
4′	3.54	71.88	3.48	71.4	−0.06	−0.48
5′	3.03	70.42	3.45	71	0.42	0.58
6′	4.89	92.13	4.28	87.9	−0.61	−4.23
7′	4.82	–	4.75	–	−0.07	–
8′	4.7	–	4.73	–	0.03	–
9′	4.52	–	4.61	–	0.09	–
10′	6.23	–	6.32	–	0.09	–
11′	4.41	–	–	–	–	–

**Table 4:** ^1^H NMR and ^13^C NMR chemical shifts (ppm) of LasX in chloroform.

No. of Atom	X		LasX			
	^1^H	^13^C	^1^H	^13^C	Δ^1^H	Δ^13^C
1′	3.44	62.5	4.28	67.9	0.84	5.4
2′	4.53	72.2	3.83	71.5	−0.7	−0.7
3′	3.45	70	3.66	71.5	0.21	1.5
4′	3.53	72.2	3.48	71.4	−0.05	−0.8
5′	3.44	62.5	3.45	71	0.01	8.5
6′	1.32	–	1.27	–	−0.05	–
7′	2.09	–	1.94	–	−0.145	–
8′	1.48	–	1.41	–	−0.069	–
9′	3.41	–	3.84	–	0.431	–
10′	1.47	–	–	–	–	–

**Table 5:** Heat of formation (HOF, kJ/mol) of LasGlu and LasX and their complexes with various monovalent cations calculated by PM6 method.

Complex	HOF (kJ/mol)	ΔHOF
LasGlu	−2742.24	–
LasX	−2590.95	
LasGlu Li^+^ (complexed)	−2495.57	−368.47
LasGlu Li^+^ (uncomplexed)	−2127.10	
LasGlu Na^+^ (complexed)	−2611.59	−422.41
LasGlu Na^+^ (uncomplexed)	−2197.16	
LasGlu K^+^ (complexed)	−2394.05	−150.36
LasGlu K^+^ (uncomplexed)	−2285.87	
LasX Li^+^ (complexed)	−2256.91	−281.10
LasX Li^+^ (uncomplexed)	−1975.81	
LasX Na^+^ (complexed)	−2369.08	−323.21
LasX Na^+^ (uncomplexed)	−2045.87	
LasX K^+^ (complexed)	−2278.80	−144.22
LasX K^+^ (uncomplexed)	−2134.58	

## Data Availability

The data is available on request.
